# Retraction: Structural characterization of centipede oligopeptides and capability detection in human small cell lung carcinoma: inducing apoptosis

**DOI:** 10.1039/d2ra90036j

**Published:** 2022-04-27

**Authors:** Laura Fisher

**Affiliations:** Royal Society of Chemistry Thomas Graham House, Science Park, Milton Road Cambridge CB4 0WF UK advances-rsc@rsc.org

## Abstract

Retraction of ‘Structural characterization of centipede oligopeptides and capability detection in human small cell lung carcinoma: inducing apoptosis’ by JingQuan Zhao *et al.*, *RSC Adv.*, 2019, **9**, 10927–10936, https://doi.org/10.1039/C8RA09018A.

The Royal Society of Chemistry hereby wholly retracts this *RSC Advances* article due to a significant amount of unattributed text overlap throughout the article, and particularly with ref. 1 in the Results and discussion section and ref. 2 in the Conclusion section.

Jie Liu opposes the retraction. The other authors have been informed but have not responded to any correspondence regarding the retraction.

Signed: Laura Fisher, Executive Editor, *RSC Advances*

Date: 29^th^ March 2022

## Supplementary Material
